# Subtype prediction in pediatric acute myeloid leukemia: classification using differential network rank conservation revisited

**DOI:** 10.1186/s12859-015-0737-3

**Published:** 2015-09-23

**Authors:** Askar Obulkasim, Maarten Fornerod, Michel C. Zwaan, Dirk Reinhardt, Marry M. van den Heuvel-Eibrink

**Affiliations:** 1 Department of Pediatric Oncology/Hematology, Erasmus-MC Sophia Childrens Hospital, Rotterdam, The Netherlands; 2 Dutch Children’s Oncology Group, Erasmus-MC Sophia Children’s Hospital, Rotterdam, The Netherlands; 3 AML-BFM Study Group, Pediatric Hematology/Oncology, Essen, Germany; 4 Princess Máxima Center for Pediatric Oncology, Utrecht, The Netherlands

**Keywords:** Relative expression, Pathways, Classification, Batch effect, High-dimensional data

## Abstract

**Background:**

One of the most important application spectrums of transcriptomic data is cancer phenotype classification. Many characteristics of transcriptomic data, such as redundant features and technical artifacts, make over-fitting commonplace. Promising classification results often fail to generalize across datasets with different sources, platforms, or preprocessing. Recently a novel differential network rank conservation (DIRAC) algorithm to characterize cancer phenotypes using transcriptomic data. DIRAC is a member of a family of algorithms that have shown useful for disease classification based on the relative expression of genes. Combining the robustness of this family’s simple decision rules with known biological relationships, this systems approach identifies interpretable, yet highly discriminate networks. While DIRAC has been briefly employed for several classification problems in the original paper, the potentials of DIRAC in cancer phenotype classification, and especially robustness against artifacts in transcriptomic data have not been fully characterized yet.

**Results:**

In this study we thoroughly investigate the potentials of DIRAC by applying it to multiple datasets, and examine the variations in classification performances when datasets are (i) treated and untreated for batch effect; (ii) preprocessed with different techniques. We also propose the first DIRAC-based classifier to integrate multiple networks. We show that the DIRAC-based classifier is very robust in the examined scenarios. To our surprise, the trained DIRAC-based classifier even translated well to a dataset with different biological characteristics in the presence of substantial batch effects that, as shown here, plagued the standard expression value based classifier. In addition, the DIRAC-based classifier, because of the integrated biological information, also suggests pathways to target in specific subtypes, which may enhance the establishment of personalized therapy in diseases such as pediatric AML. In order to better comprehend the prediction power of the DIRAC-based classifier in general, we also performed classifications using publicly available datasets from breast and lung cancer. Furthermore, multiple well-known classification algorithms were utilized to create an ideal test bed for comparing the DIRAC-based classifier with the standard gene expression value based classifier. We observed that the DIRAC-based classifier greatly outperforms its rival.

**Conclusions:**

Based on our experiments with multiple datasets, we propose that DIRAC is a promising solution to the lack of generalizability in classification efforts that uses transcriptomic data. We believe that superior performances presented in this study may motivate other to initiate a new aline of research to explore the untapped power of DIRAC in a broad range of cancer types.

**Electronic supplementary material:**

The online version of this article (doi:10.1186/s12859-015-0737-3) contains supplementary material, which is available to authorized users.

## Background

High-throughput genomic technologies have dramatically expanded the breadth of biological information available for the analysis and characterization of disease. One of the most important application fields of transcriptomic data is classification, where a multitude of promising results have been reported in classification literatures with near perfect classification accuracy for given datasets. Yet, despite this progress, the reliability of these results has been proven illusory, as the seemingly promising results often fail in reproducing, inextricable yet largely unachieved goal, in new datasets [1]. This is mainly due to the fact that a single transcriptome contains tens of thousands of features, while a limited number of samples are available for analysis. A large number of redundant, irrelevant features and technical noise added during the data generation process make classifiers susceptible to over-fitting [2]. In some cases, even a small change in data preprocessing can lead to substantial changes in downstream analyses [3]. The obvious and ideal remedy of increasing the sample size is often infeasible, leaving researchers in a quandary about how to increase the models’ reliability. Thus, the search for a robust classifier in clinical application becomes a notoriously difficult endeavour.

In recent years, there has been a burgeoning interest in system methods that incorporate network information into classification algorithms for biomarker discovery in personalized medicine [4–7]. The general hope is that the application of hard-earned biological domain knowledge can improve the typical low reproducibility of the biomarkers. A promising example, the topic of this article, is the Differential Rank Conservation (DIRAC) algorithm proposed by Eddy *et al.* (2010) [4].

Here, our intention is not to re-introduce DIRAC, but rather to explore some of the undocumented characteristics of a DIRAC classifier in common genomic classification scenarios. We aim to broaden researchers’ familiarity with the pros and cons of DIRAC, especially in identifying robust cancer subtype specific network signatures that are robust against unwanted variations stemming from differences in preprocessing, platforms, batches, and other confounding variables.

## DIRAC overview

DIRAC is a member of a family of algorithms that work with the relative expression of genes [8, 9]. Since DIRAC uses the relative rather the absolute expression of genes for disease classification, algorithms fall in the latter category populated the journal landscape, it has potentials to be robust against ancillary sources of variation in high-dimensional molecular data.

Philosophy of DIRAC based classifier can be summarized as follows:

- It tried to capture the biological system as a whole instead of reporting a list of individual parts as the conventional classifier does.
- Focus on genesets (pathways) as opposed to individual genes. Hence, valuable gene-gene interaction could be harvested.
- Resulting genesets (pathways) from the DIRAC based classifier is more intuitive to biologist.

**Fig. 1:**
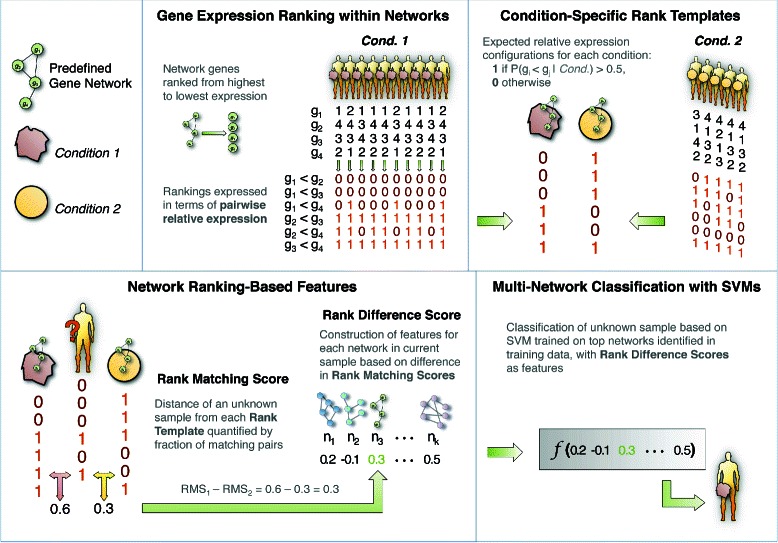
Overview of DIRAC method for constructing multi-network classifier

DIRAC is composed of a set of measures to quantify differential expression variability between conditions using subsets of genes. These subsets generally correspond to predefined gene networks or pathways. The rationale for composing the analysis using these subsets comes from the perspective of systems biology. The contextualization of expression changes in functional units is viewed as more informative than looking at gene-level changes in isolation. Briefly, DIRAC transforms the rank ordering of genes within a network into a binary vector of pairwise gene comparisons (Fig. 1). A more complete discussion of DIRAC can be found in Eddy *et al.* (2010) [4]. Each element of the Rank Template is 1 if the probability of the gene comparison at that position else 0, i.e., *v*_*ij*_= [*g*_*i*_<*g*_*j*_]. This binary vector is then used to create a most likely binary vector termed the Rank Template. Each element of the Rank Template is 1 if the probability of the gene comparison at that position is greater than 50 % under a given condition, i.e., *R* *T*_*ij*_= [ *P*(*g*_*i*_<*g*_*j*_|*C*)>0.5]. The Rank Template for each condition is then used in classification to generate a Rank Matching Score. This is effectively one minus the Hamming distance between an unknown sample’s pairwise gene comparison and the Rank Template, i.e., $RMS = \sum {[\!RT_{\textit {ij}} = v_{\textit {ij}}]}/|RT|$. Traditionally, classification can then be done by comparing distances, and classifying the unknown sample as the condition with the closest Rank Template. In this study, DIRAC is extended to multiple networks by using the vector of Rank Difference Scores (i.e., $RMS_{C_{1}} - RMS_{C_{2}}$) as a feature vector in a pre-trained Support Vector Machine. This formulation also allows us to trivially and robustly extend DIRAC to multi-class 1-vs.-Rest classification.

We find it necessary to point out that the DIRAC is *not* a classification algorithm. It falls within the realm of data transformation, i.e. project samples that are originally in the gene space to a much lower dimensional pathway space. The hope is that the difference between groups under investigation will be pronounced in the new space. The transformed data can be combined with any exiting classification algorithm.

## Results and discussion

We first perform the pathway and GEV signatures identification for each cytogenetic subtype. Then, the signatures are used in later stage to evaluate their robustness against different preprocessing techniques and reproducibility in independent datasets in presence of batch effect, respectively. Performances of the two classifiers are measured by calculating their sensitivity, specificity and F-score. The F-score, that equals 2(*s* *e* *n* *s* *i* *t* *i* *v* *i* *t* *y*×*s* *p* *e* *c* *i* *f* *i* *c* *i* *t* *y*)/(*s* *e* *n* *s* *i* *t* *i* *v* *i* *t* *y*+*s* *p* *e* *c* *i* *f* *i* *c* *i* *t* *y*), is a harmonic mean of the former two. It ranges from 0 to 1, with one being the perfect classification.

### Signature identification for cytogenetic subtypes using the pediatric ^1^ AML dataset.

**Fig. 2:**
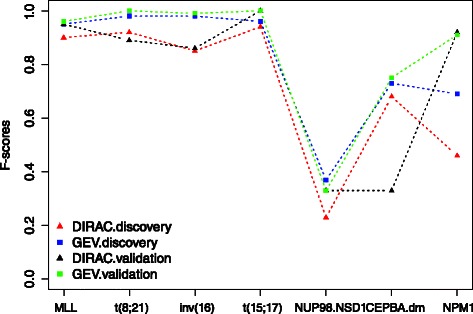
Classification performances of the DIRAC-based and GEV-based classifiers on the pediatric ^1^ AML dataset. The x-axis shows the cytogenetic subtypes present in the data, the y-axis shows the classification accuracy quantified as F-score. For the DIRAC.discovery and GEV.discovery each value represents the mean of 100 OCV

The pediatric ^1^ AML dataset was used to identify both gene expression and pathway signatures for each cytogenetic subtype. The mean prediction accuracy of 100 OCV iterations from the gene expression based and the DIRAC-based classifiers are shown in Fig. 2. The prediction accuracies on the validation set with the optimal signature lists are also given. We observed that the GEV-based classifiers render higher prediction sensitivities both on the discovery and the validation sets compared to the DIRAC-based classifiers. The specificities from two approaches, however, are comparable.

#### GEV signatures

- **MLL**: C10orf140, DEXI, HOXA7.
- **t(8;21)**: RUNX1T1, POU4F1, CACNA2D2.
- **inv(16)**: MN1, TM4SF1, MYH11, CLIP3, SPARC, AK5, PTRF, FAM171A1, AK022033, LOC100506388.
- **t(15;17)**: STAB1, FGF13, LGALS12, PTGDS.
- **NUP98.NSD1**: VENTX, PRDM16, FAM92A1.
- **CEPBA.dm**: LRRC28, CNRIP1, ST8SIA1, MSS51, SLC14A1, LGSN.
- **NPM1**: HOXB-AS3, HOXB3, SMC4, SEPP1, LTBP1, CLIC2, HOXB4, TWISTNB, HIST1H2BC, SIAE, PIEZO2, HLF, ELL2, LOC100507520, HOXB2, FOXC1, HOXB6, HOXB5, EMR1, SNCAIP, RPL39L, USP44, BEX1, TTC27, PTPRC, HENMT1, AK027199, COL4A5, NAP1L5, TIAM1, NPDC1, CLEC11A, SCD5, CCL1, FTO, AK093529, ENSG00000184551, CDKN1B, FAM105A, PHLDA1, HOXA-AS5, LOC100506591, GMDS, TOM1L1, IL12A, DMXL2, SDPR, FOXF1.

DIRAC considers a pathway to be tightly regulated under a given condition if, for that condition, the rank ordering of the genes in that pathway are highly correlated; loosely regulated if they are less correlated. We noticed that, the pathway signatures (Table 1) specific to AML subtype represent salient signal transduction pathways with known roles in cell proliferation and/or differentiation. In particular, it is noteworthy that the subgroups characterized by inv(16), t(15;17) and *CBPA* double mutations, *NPM1* mutations are characterized by one or more tightly regulated MAP kinase pathways, P38MAPK, KERATINOCYTE and/or MAPK. This suggests that inhibition of MAP kinase activity within these pathways may be a way to effectively target these cells, which needs further biological validation studies. Another pathway of interest is the BIOPEPTIDES (Bioactive Peptide Induced Signaling) pathway, which is characterized by the RAS, JAK and STAT signal transduction route. This pathway appears to be tightly regulated in subgroups characterized by t(8;21), Inv(16) and NUP98/NSD1. Surprisingly, AML with *MLL* rearrangements were not classified by any tightly regulated pathway, but rather by three loosely regulated pathways. This may be explained by the relative heterogeneity of this subgroup, which contains several *MLL* translocation partners, in particular AF6, AF9 and AF10, which have strikingly different clinical characteristics [10].

**Table 1:** The rank matching scores (RMS) (see Fig. 1) of the signature pathways generated by the DIRAC-based classifier

Pathways	MLL	t(8;21)	inv(16)	t(15;17)	NUP98.NSD1	CEPBA.dm	NPM1
AGR							
ALK							
AT1R							
ATRBRCA							
BCR							
BIOPEPTIDES							
CELLCYCLE							
CHREBP2							
EIF							
FCER1							
GPCR							
INTEGRIN							
KERATINOCYTE							
MAPK							
NO1							
P38MAPK							
PPARA							
TNFR1						0.000	

In each column, numbers denote the difference between the mean RMS of samples in a particular subtype and the mean RMS of remaining samples (e.g. RMS _*MLL*_ - RMS _*Rest*_). Numbers in red denote the pathway is tightly regulated in a subtype compared to the test, and opposite applies to the pathway in blue

These pathway signatures may be options for understanding specific disease mechanisms and provide a key for the design of new treatment. For example, one may seek network aware intervention to adjust the signature pathways in each subtype to its normal non-leukemic cell behavior and, consequently, to control clinical presentation.

### Assessing the robustness of the DIRAC-based and GEV-based classifiers against different preprocessing techniques

Multiple preprocessing methods for gene expression data have been proposed [11]. However, none of them are reported to be uniformly better than the other. Schmidt *et al.* (2011) [3] reported that findings in gene expression data analysis significantly depend on the preprocessing method used. Here, we examined the effect of a mundane but indispensable task of ‘data normalization’ on classification. We compared the robustness of the two competing approaches when the data was preprocessed with different methods. Specifically, a classifier was trained with the pathway and gene expression signatures obtained from the pediatric ^1^ dataset and were used to predict subtype labels of the same dataset that has been preprocessed using six well-known methods. This is meant to be representative of a situation where a researcher intends to combine or compare transcriptomic profiles from two studies where the preprocessing methods were different and the original unprocessed data is not available. Ideally, one would have the original data and use the same preprocessing methods on those data sources, but this is not always an option. The six preprocessing methods are: M1) CEL file transformed to raw expression data using Robust multi-array average expression measure (RMA); M2) Raw data obtained with RMA and normalized with quantile normalization; M3) CEL file transformed to raw expression data using Affymetrix’s MAS 5.0 expression measure (MAS5); M4) Raw data obtained with MAS5 and normalized with scale normalization; M5) CEL file transformed to raw expression data using Robust multi-array average expression measure with help of probe sequence (GCRMA); M6) Raw data obtained with GCRMA and normalized with quantile normalization.

**Fig. 3:**
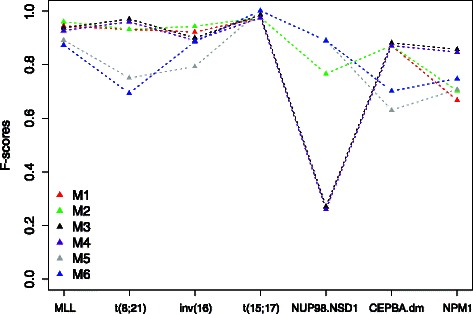
Evaluation of the robustness of the pathway signatures generated by the DIRAC-based classifier on the pediatric ^1^ dataset that has been underwent different types of preprocessing. The x-axis shows the cytogenetic subtypes in the data, the y-axis shows the classification accuracy quantified as F-score

**Fig. 4:**
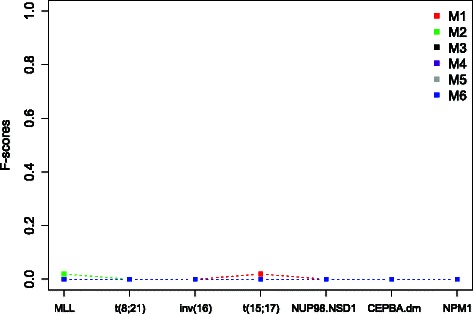
Evaluation of the robustness of the gene signatures generated by the GEV-based classifier on the pediatric ^1^ dataset that has been underwent different types of preprocessing. The x-axis shows the cytogenetic subtypes in the data, the y-axis shows the classification accuracy quantified as F-score

Figures 3 and 4 display results from the two classifiers in the aforementioned six scenarios. Apparently, the DIRAC-based classifier was largely invariant against the preprocessing methods. The highest variations were observed in the NUP98.NSD1 and CEPBA.dm subtypes. We believe this mainly due to small sample sizes. The gene expression based classifier was, on the other hand, very sensitive to preprocessing method. In practice the latter appears to be of limited use.

### Assessing the robustness of the DIRAC-based and GEV-based classifiers against batch effect

Genomic data often contain batch effects, i.e. differences in sample sources and other technical variations. Many algorithms have been proposed to remove these artifacts, but this (often) comes along with the potential cost of removing between-group biological heterogeneity and consequently salient genomic signatures [12]. Note that, in this work we refer to the batch effect as differences between datasets that have: generated in different platforms or different labs, included in two different studies.

**Fig. 5:**
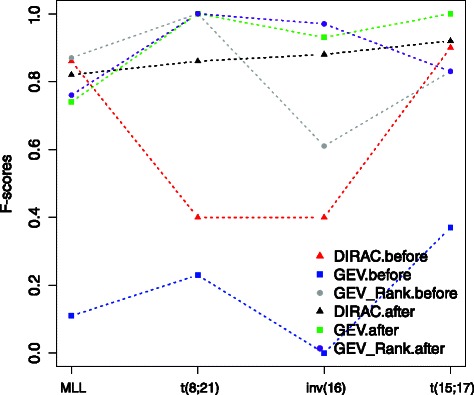
Validation of the previously obtained subtype signatures (gene and pathways) using the pediatric ^2^ AML dataset. The validation is performed with batch effect present and batch effect corrected cases, separately. Accuracies from the DIRAC, GEV, and GEV Rank based classifiers are shown for each subtype. The x-axis shows the cytogenetic subtypes, the y-axis shows the classification accuracy quantified as F-score

**Fig. 6:**
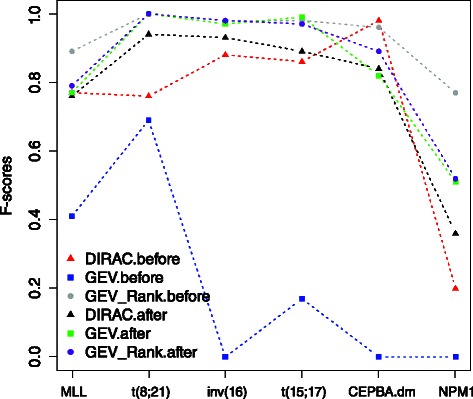
Validation of the previously obtained subtype signatures (gene and pathways) using the adult AML dataset. The validation is performed with batch effect present and batch effect corrected cases, separately. Accuracies from the DIRAC, GEV, and GEV Rank based classifiers are shown for each subtype. The x-axis shows the cytogenetic subtypes, the y-axis shows the classification accuracy quantified as F-score

To demonstrate the robustness in translating trained predictor across independent datasets, we analyzed performance characteristics of the DIRAC-based classifier as well as the GEV-based classifier on the pediatric ^2^ and the adult AML datasets. The GEV-based classifier relies on expression values and reflects commonly used practice when no additional knowledge is available. Both pathway and gene expression classifiers were trained using all samples in the pediatric ^1^ dataset and used to predict the subtype labels of the two independent datasets. We observed strong batch effects between the pediatric ^1^ and the two new independent datasets (see Figure S1 and Figure S2 in the Additional file 1). We tested our trained classifiers against these datasets both with and without batch effect corrections [13]. Results from these two scenarios are shown in Figs. 5 and 6. In both datasets, we observed that the stellar performance of the GEV-based classifier disappeared in the presence of batch effects. Without batch correction, the classifier was completely ‘confused’, as reflected by the fact that it classified all new samples as a single category, resulting in extremely poor specificity. The performance of the classifier improved significantly after batch correction in both datasets. The DIRAC-based classifier exhibited stable performances in the adult AML dataset, and in the pediatric ^2^ AML dataset its showed similar performances only in the *MLL* and t(15;17) subtypes.

In order to better comprehend the prediction power of the DIRAC-based classifier in general, we conducted performance comparison using publicly available datasets from breast and lung cancer (see Additional file 1). Also, multiple well-known classification algorithms were utilized to create an ideal test bed for comparing the two competing approaches. We obtained two indenpedent breast cancer datasets with substantial batch-effect. Similar to the classification settings mentioned above, we used one of them used for signature discovery (discovery set) and the second one for testing the signature reproducibilty (test set). We observe that the DIRAC classifier performed similarly in the discovery set, but greatly outperformed the standard GEV-based classifier in test set without batch effect correction. These results corroborate our findings in the AML datasets.

### Where does the power of the DIRAC-based classifier come from?

**Fig. 7:**
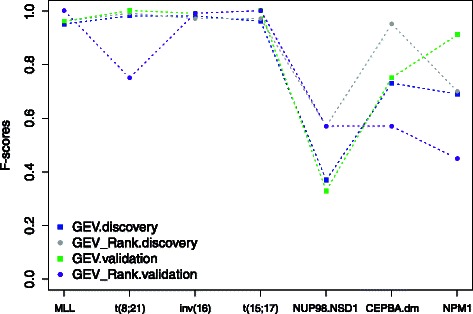
Performances comparison between the GEV rank-based and GEV-based classifiers using the pediatric ^1^ AML dataset. The x-axis shows the cytogenetic subtypes present in the data, the y-axis shows the classification accuracy quantified as F-score. For the GEV.discovery and GEV_Rank.discovery each value represents the mean of 100 OCV

**Fig. 8:**
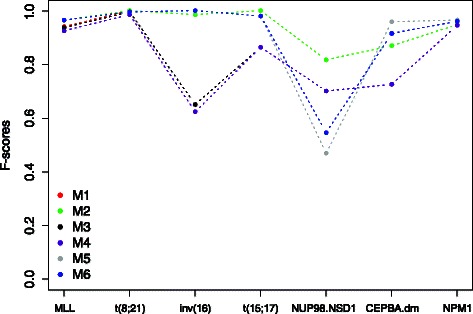
Evaluation of the robustness of the gene signatures generated by the GEV_Rank-based classifier on the pediatric ^1^ dataset that has been underwent different types of preprocessing. The x-axis shows the cytogenetic subtypes, the y-axis shows the classification accuracy quantified as F-score

To investigate whether the more stable cross-batch performance of the DIRAC-based classifier stems from the pathway information used or due to the use of relative expression, we repeated aforementioned analyses only using genes’ relative expressions without pathway data. Specifically, in each sample we ranked genes according their expression values (low to high). Then, the rank matrix, instead of the original of gene expression values, was used to construct classifiers. Classification results are shown in Fig. 7. We observed that the rank-based classifier produced comparable results with the DIRAC-based classifier, and in some subtypes, even better. Based on these results, we concluded that more robust performances of the DIRAC-based classifier in these subtypes are seemingly attributed to its nature of rank-based measures. This makes it invariant to batch correction (Figs. 5–6) and preprocessing techniques (Fig. 8) that leave the relative ranks unchanged, while many other techniques that use absolute gene expression values are obviously sensitive to 5. The similar performance between using ranks directly and DIRAC is unsurprising given that recent studies have shown that DIRAC is strongly related to (and trivially transformed to) Kendall’s Tau rank correlation statistic [14].

## Conclusion

Clearly, the DIRAC-based classifier is robust against most of the major ancillary sources of variation in the data. We attribute this to the robustness of relative expression compared to absolute expression values used [15] and the ability to tap the orchestrated behaviour of genes within transcriptome using the domain knowledge.

According to the no-free-lunch theorem [16] no classification algorithm is uniformly better than the others. Hence, we do not claim that DIRAC is a panacea for all types of genomic classification problems. As we showed in this study, there are cases in which the GEV-based classifier outperforms the DIRAC-based classifier. The focal point is, however, placed on the robustness of the latter. We recommend the DIRAC-based classifier when the choices of preprocessing and batch effect correction methods are not obvious, either by limited statistical resources or due to limited data availability. It is, for example, often the case that raw data (e.g. CEL files) are not publicly available. Thus, albeit numerous databases exist to store tremendous amounts of genomic data, it is surprisingly difficult to find a dataset that has been preprocessed in exactly the same way as the one from which new findings (e.g. subtype signatures) were discovered and are in need to be validated. As we demonstrated in this study, these issues, to a large extent, are ameliorated when the DIRAC-based classifier is used. To our surprise, the trained DIRAC-based classifier even translated well to a dataset with different biological characteristics (e.g. adult AML) in the presence of substantial batch effects that, as shown here, plagued the standard GEV-based classifier. In addition, the DIRAC-based classifier, because of the integrated biological information, also suggests pathways to target in specific subtypes, which may enhance the establishment of personalized therapy in diseases such as pediatric AML.

In this study, we investigate the performance of the DIRAC-based classifier using multiple AML datasets. We believe that its superior performances presented in this study may motivate other to explore the untapped power of DIRAC in different types of cancer or may even open a new line of application.

In summary, through this study we have demonstrated the robustness of DIRAC in classification, which has previously been undocumented and underestimated. While this is not the first multi-class DIRAC method proposed [17], we have demonstrated the first multi-network classification method using DIRAC. We believe that the robustness, simplicity and biological interpretability of the DIRAC-based classifier make it not only an attractive alternative to existing algorithms, but often a preferred choice.

## Methods

In this study we investigate the potentials of DIRAC in multiple scenarios frequently appear in analyzing high-dimensional transcriptomic data. The number of conceivable applications of DIRAC is possibly large, hence we do not claim to provide exhaustive list. Instead, the focus is on classification problem that has been the subject of bioinformatics research for long time. Reported experimental results from multiple real-world datasets serve two ends: 1) to illustrate the benefits of the DIRAC-based classifier, 2) to clarify what makes the DIRAC-based classifier superior than exiting approaches. In the following sections we describe the datasets that are considered in this study, and the experimental setups, i.e. how we compare performance of the DIRAC-based classifier with exiting approaches in an unbiased way.

### Gene expression datasets

Three publicly available acute myeloid leukemia (AML) datasets (Table 2) were used to assess the performance of the DIRAC-based classifier, and compare it with the traditional gene expression value (GEV) based classifier. The three AML gene expression datasets of well-characterized cytogenetic and molecular subtypes of AML are depicted in Table 3. In this study, samples were limited to those with one of seven major cytogenetic subtypes [18]. For the pediatric ^1^ AML dataset, which was generated in our lab, the raw data (CEL files) were also available [18]. Details of the datasets and preprocessing are given in the Additional file 1. In each dataset, the probe-level data are transformed into the gene-level by taking the mean of probes that interrogate a single gene on which downstream analyses were performed.

**Table 2:** Summary of AML datasets used in this study

Dataset	Reference	# Samples	Usage
Pediatric ^1^ AML	Balgobind et al. 2011 [18]	199	Signature identification
Pediatric ^2^ AML	Radtke et al. 2009 [21]	58	Signature validation
Adult AML	Verhaak et al. 2009 [22]	323	Signature validation

**Table 3:** Cytogenetic and molecular characteristics of the AML datasets included in this study

Cytogenetic subtypes	Pediatric ^1^ AML	Pediatric ^2^ AML	Adult AML
MLL	68	15	34
t(8;21)	30	20	38
inv(16)	35	16	42
t(15;17)	20	7	25
NUP98.NSD1	13		
CEPBA.dm	13		26
NPM1	20		158

### Pathway source

In this study, 217 manually curated (comprised of 1279 unique genes) BioCarta pathways from the Molecular Signatures Database (MSigDB version 4.0, updated May 31, 2013) were used to construct the DIRAC-based classifier. For each dataset, genes were grouped according to their presence in these pathways. To reliably represent the pathways, only those pathways that have at least 30 % of associated genes presented in a given dataset were retained. The minimum fraction of genes that are present both in individual pathways and gene expression data is: 70 % in the pediatric ^1^ and adult AML datasets, 57 % in the pediatric ^2^ AML dataset. Note that, for the DIRAC-based classifier only those genes that belong to the retained pathways were used. For the GEV-based classifier the full list of genes, not just the pathway mapped subsets, was used. Note that, the number of genes used for the GEV-based classifier is far more exceeds the number of pathways used for the DIRAC-based classifier. This means, the former has a larger search space to find the discriminative features compared to the latter.

### Classifier construction and signature identification

We used the pediatric ^1^ AML dataset (Table 2) to generate gene (pathway) signatures specific to each cytogenetic subtype (i.e., via 1-vs.-Rest multiclass classification scheme) using Support Vector Machines (SVM). Our strategy to extract both signatures is similar to the one used in Balgobind *et al.* (2011) [18]. Specifically, the dataset is randomly divided, subject to stratification that maintains the proportionality of the subtypes, into a discovery set (2/3 samples) and validation set (1/3 samples). The discovery set is used to select the subtype specific signatures, and the validation set serves as independent validation cohort. Importantly, the validation set is not used at any point in classifier training.

**Fig. 9:**
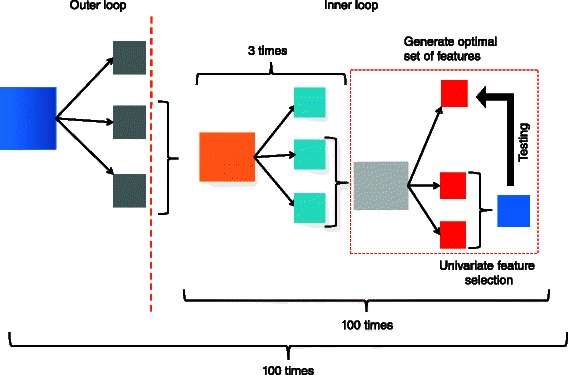
Schematic of the classifier construction and signature identification

To obtain reliable results, we performed 100 outer-cross-validation (OCV) and 100 inner-cross-validation (ICV) on the discovery set (Fig. 9). In each of 100 OCV iterations the following steps were taken: the discovery set was further randomly divided into a training set (2/3 samples) and a test set (1/3 samples). Then, 100 ICV iterations were performed on the training set. In each of 100 ICV iterations the training set was further divided, again subject to stratification constraints, into an inner-training (2/3) and an inner-test (1/3) set. We applied an empirical Bayes linear regression model [19] on the inner-training set to select top 50 features that discriminate each AML subtype under consideration from the rest.

The selected features (50 genes/pathways) were processed in following ways to obtain reduced set of GEV and pathway signatures. For each subtype, the GEV (pathways) signatures were ranked (from best to worst) according to their association with the subtype via the global test [20]. Subsequently, an SVM classifier was trained (e.g. MLL vs. Rest) using the 50 genes (pathways) and predictive performance on the inner-test set was gauged. The size of the signature list was then reduced from 50 by removing the gene located at the bottom of the list of ranked genes (pathways), and the classification was re-run. This process was repeated until there were no more than 3 genes (pathways) remaining and mean sensitivity (across subtypes) was recorded. Finally, the list that corresponds to the median sensitivity over the 3 ICV train-test procedures is chosen.

At the end of 100 ICV iterations, a signature list of smallest size that renders highest prediction sensitivity was determined. Among the three optimal lists generated via 3-fold CV using the discovery set, the one with the median prediction sensitivity was taken as the optimal signature list for one OCV iteration. The final classifier was trained using the signature list that produced the highest prediction sensitivity in 100 OCV iterations and subsequently used to predict the labels of the validation set. Balgobind *et al.* (2011) [18] argued that this double-loop CV avoids over-fitting and leads to stable signatures with highest prediction accuracy.

### Ethics approval

Ethics approval is not required for this study.

## Additional file

Additional file 1 **Supplementary material.** This document includes additional information not included in the paper. (PDF 1239 kb)
