# Supplementary material for: Subtype prediction in pediatric acute myeloid leukemia: classification using differential network rank conservation revisited
Source: BMC Bioinformatics. 2015 Sep 23;16:305. doi: 10.1186/s12859-015-0737-3 (PMC4580220; doi:10.1186/s12859-015-0737-3)
Supplement: Supplementary file 1 — Supplementary material. This document includes additional information not included in the paper. (PDF 1239 kb) [file 12859_2015_737_MOESM1_ESM.pdf]

Subtype prediction in pediatric acute myeloid leukemia: classification using differential network rank conservation revisited

Supplementary document

This document provides supplementary information not mentioned in the paper.

1 Details of the leukemia datasets used in the paper and pre-processing procedures

1.1 The pediatric[1] AML dataset

- **Cancer type:** Pediatric acute myeloid leukemia (AML).
- **Molecular level:** Gene expression.
- **Reference:** Balgobind *et al.* (2011)
- **Array platform:** Affymetrix Human Genome U133 Plus 2.0 Array.
- **Number of samples:** 199.
- **Groups to classify:** Cytogenetic subtypes: ALL(n = 68), t(8;21)(n = 30), inv(16)(n = 35), t(15;17)(n = 20), NUP98.NSD1(n = 13), CEPBA.dm(n = 13) and NPM1(n = 20).
- **Availability:** Gene Expression Omnibus, accession number GSE17855.
- **Preprocessing:** CEL files were transformed to probe intensity data using the *rma* function in the R-package "affy". Probe sets were underwent filtering according to the following criteria: a probe either interrogates only one gene or specificity of the probe (available in the Weizmann Institute Affymetrix probe-sets annotation database, `http://genecards.weizmann.ac.il/geneannot/index.shtml`) is at least 0.8. This led to a data of 39634 probe sets. Then, the reduced probe level data were converted into a gene level data by collapsing those probes that interrogate the same gene to their mean. The gene level data that comprised of 22564 genes were normalized using the 'vsn' function in the R-package "VSN".

1.2 The pediatric[2] AML dataset

- **Cancer type:** Pediatric acute myeloid leukemia (AML).
- **Molecular level:** Gene expression.
- **Reference:** Radtke *et al.* (2009).
- **Array platform:** Affymetrix Human Genome U133A Array.
- **Number of samples:** 58.
- **Groups to classify:** Cytogenetic subtypes: ALL(n = 15), t(8;21)(n = 20), inv(16)(n = 16) and t(15;17)(n = 7).
- **Availability:** Gene Expression Omnibus, accession number GSE14471.
- **Preprocessing:** CEL file were transformed to probe intensity data using Microarray Suite version 5.0 (MAS 5.0) in the R-package "affy" with Affymetrix default analysis settings and subsequently 'vsn' normalization was applied.

The adult AML dataset

- **Cancer type:** Adult acute myeloid leukemia (AML)
- **Molecular level:** Gene expression.
- **Reference:** Veerhaak,R.G. *et al.* (2009).
- **Array platform:** Affymetrix Human Genome U133 Plus 2.0 Array.
- **Number of samples:** 323.
- **Groups to classify:** Cytogenetic subtypes: ALL(n = 34), t(8;21)(n = 38), inv(16)(n = 42), t(15;17)(n = 25), CEPBA.dm(n = 26) and NPM1(n = 158).
- **Availability:** Gene Expression Omnibus, accession number GSE6891.
- **Preprocessing:** CEL file were transformed to probe intensity data using Microarray Suite version 5.0 (MAS 5.0) in the R-package "affy" with Affymetrix default analysis settings and subsequently 'van' normalization was applied.

2 Visualization of the batch effect

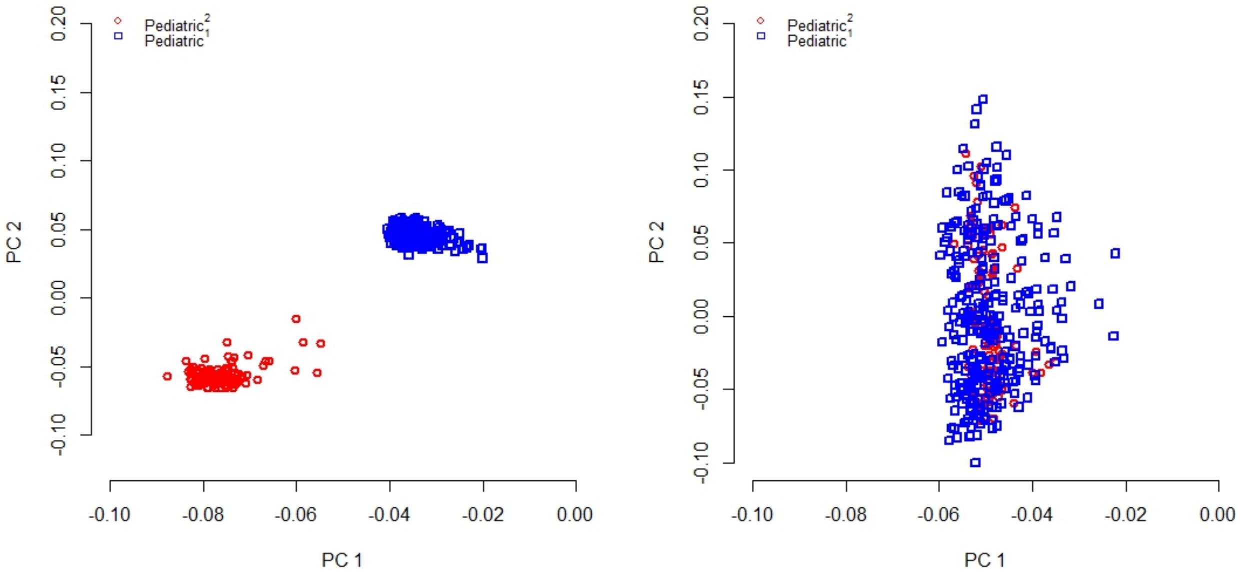

Figure S1: Visualization of the batch effect between the pediatric[1] and pediatric[2] AML datasets. The combined dataset is projected onto the space defined by the first two principal components before (left) and after (right) undergoing a batch effect correction.

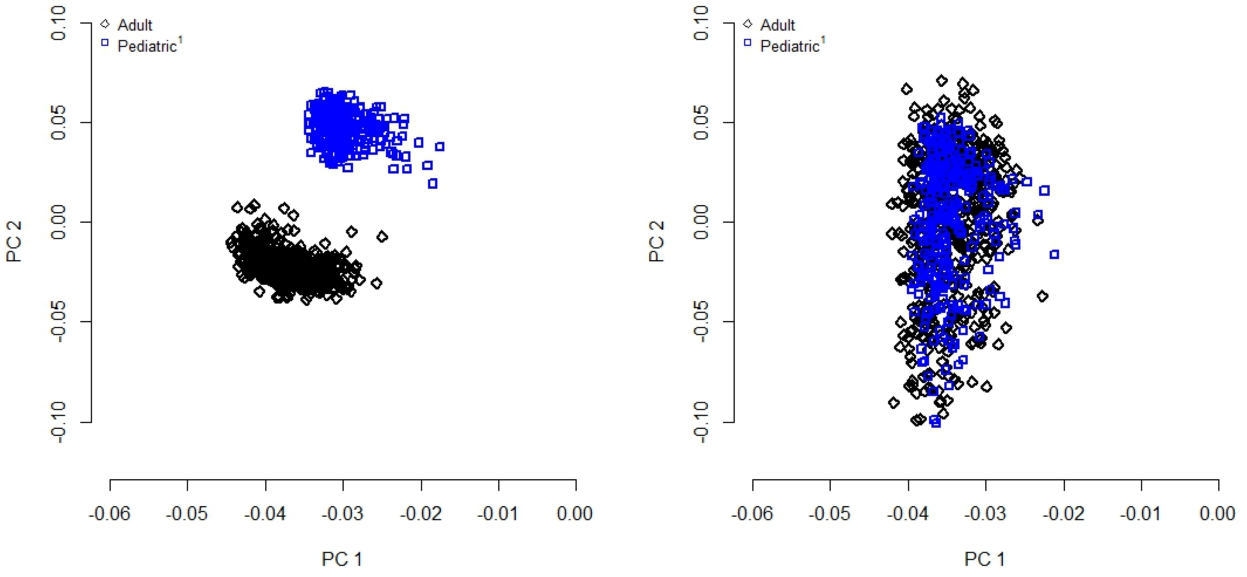

Figure S2: Visualization of the batch effect between the pediatric[1] and adult AML datasets. The combined dataset is projected onto the space defined by the first two principal components before (left) and after (right) undergoing a batch effect correction.

3 Performance comparison of the DIRAC-based and the GEV-based approaches when diverse classification algorithms are used

To support our argument that our decision to use SVM as a classifier is unbiased, in this section, we present results from the two competing approaches when three different classification algorithms, i.e. Random Forest (RF), Logistic Regression (LR) and shrunken centroid classifier (PAM), are used. For this illustration we used the pediatric[1] AML dataset. The same as in the paper, one third of the samples are used for validation. The double-loop-cross-validation is applied on the remaining samples to obtain robust signatures. Finally, the signatures are applied to the validation set and results are reported.

It can be seen from Figure S3-S5 that, variations from algorithm to algorithm are very modest. Most importantly, differences in performances of two approaches are quite stable across the algorithms. Hence, we conclude that superior performances of the DIRAC-based approach over its competitor are not due to the SVM classifier we used.

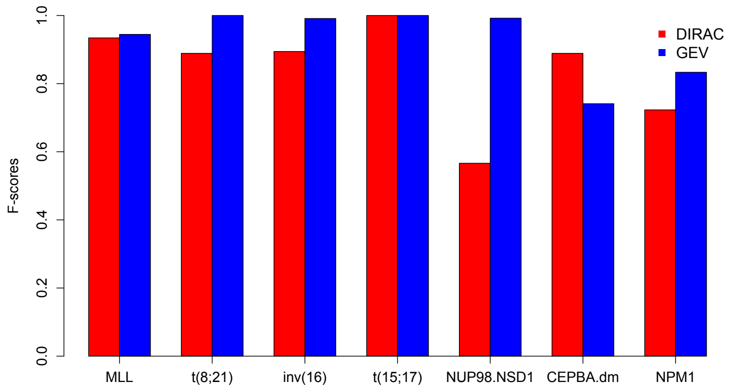

Figure S3: Classification performances of the DIRAC-based and GEV-based classifiers on the pediatric[1] AML dataset when the **LR** used as a classifier. The x-axis shows the cytogenetic subtypes, the y-axis shows the accuracy quantified as F-score. The height of each bar presents the accuracy obtained on the validation set.

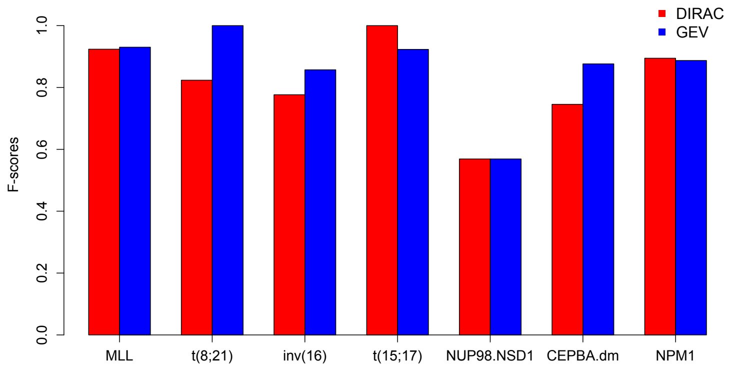

Figure S4: Classification performances of the DIRAC-based and GEV-based classifiers on the pediatric[1] AML dataset when the **PAM** used as a classifier. The x-axis shows the cytogenetic subtypes, the y-axis shows the accuracy quantified as F-score. The height of each bar presents the accuracy obtained on the validation set.

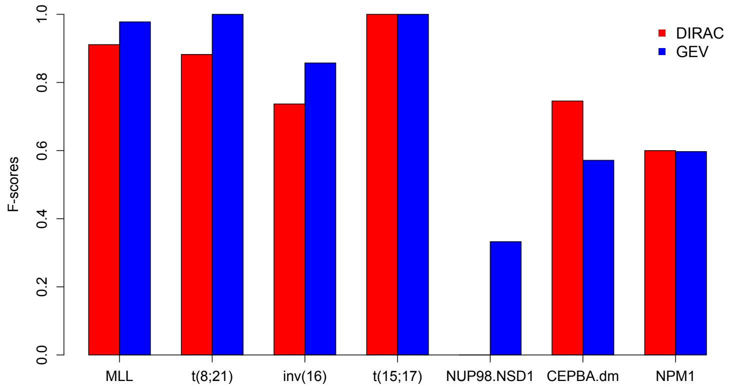

Figure S5: Classification performances of the DIRAC-based and GEV-based classifiers on pediatric[1] AML dataset when the **RF** used as a classifier. The x-axis shows the cytogenetic subtypes, the y-axis shows the accuracy quantified as F-score. The height of each bar presents the accuracy obtained on the validation set.

4 Classification performance comparison using non-leukemic datasets

To demonstrate the superiority of the DIRAC-based classifier over the standard GEV-based classifier in general, we conducted a series of performance comparisons using multiple independent non-leukemic datasets. We collected publicly available datasets from breast and lung cancer (see below). We refer to the original publications and the GEO database for patient and sample characteristics. The performances of the two approaches are compared using SVM, RF, LR and PAM algorithms, separately. Note that, in the breast cancer setting, besides the dataset that is used for signatures discovery, there is

an independent dataset to test the reproducibility of the signatures obtained by the two approaches. For the sake of clarity, we termed the first dataset as a Discovery set, the second one as a Test set. The same as in the paper, in the discovery set, one third of the samples are left out for validation purpose. The double-loop-cross-validation is applied on the remaining samples to obtain robust signatures. Finally, the signatures are applied to the Test set (if available) and results are reported. In the following sections we detail the datasets and performances of the two approaches on them.

4.1 The breast cancer dataset

Two publicly available breast cancer datasets are used for performances comparison. Two binary classifiers are constructed to discriminate the cases with oestrogen receptor positive from the negative ones. First, a dataset from Wang et al (2012) was used as a Discovery set to extract gene and pathways signatures, separately. Then, two classifiers were trained using the signatures (gene and pathways signatures, separately) on this dataset. Subsequently, the trained classifiers were applied to an independent dataset (Test set) from van de Vijver *et al.* (2002) and van't Veer *et al.* (2002). Note that, there is strong batch effect between the Discovery and Test sets (Figure S7). Similar to the experimental settings given in the paper, we evaluated the reproducibility of the signatures by applying them to the Test set, with and without a batch effect correction. The batch effect correction was performed using the 'ComBat' function in the R-package `sva`.

As we observe from Figure S7, the two competing approaches performed similarly across different classification algorithms. This may further strengthen our argument that our decision to use SVM as a primary classification tool is free of selection bias. While the DIRAC-based classifier kept similar promising performances, the GEV-based classifier pushed all samples into one class (F-score = 0) in the Test set *with* batch effect as shown in Figure S8 (left). While improved performances of the GEV-based classifier are observed (Figure S8 right), accuracies from the DIRAC-based classifier decreased substantially after batch effect correction. This example support our claim in the paper that batch effect correction sometimes do harm than good. In this particular example the batch effect correction procedure removed some important biological variations specific to each group.

The dataset used for signature discovery:

- **Cancer type:** Breast cancer.
- *Molecular levels:* Gene expression.
- **Reference:** Wang *et al.*, *Lancet*, 2005.
- **Array platform:** Affymetrix oligonucleotide microarray U133a GeneChip.
- **Number of samples:** 286.
- **Groups to classify:** Estrogen receptor status: positive (n = 209) and negative (n = 77).
- **Availability:** Gene Expression Omnibus (GEO), accession number GSE2034.

- **Usage:** Signature discovery (Discovery set).

- **Preprocessing:** we downloaded the normalized dataset that has measurements on 22283 probes. By collapsing into the gene level, dataset size reduced to 13212 genes. Subsequently, the dataset was underwent log2 transformation. No missing values were detected. To match with the size of the Test set, we kept only those genes that existed in the Test set. The final dataset contains 9786 genes.

The dataset used for testing the reproducibility of signatures from the Discovery set:

- **Cancer type:** Breast cancer.

- *Molecular levels:* Gene expression.

- **Reference:** van de Vijver *et al.*, *N Engl J Med*, 2002; van't Veer *et al.*, *Nature*, 2002.

- **Array platform:** N.A.

- **Number of samples:** 337.

- **Groups to classify:** Estrogen receptor status: positive (n = 249) and negative (n = 88).

- **Availability:** The R-package "breastCancerNKI".

- **Usage:** Evaluate signature reproducibility (Test set).

- **Preprocessing:** We obtained the normalized dataset that has measurements on 24481 probes from the R-package "breastCancerNKI". By collapsing into the gene level, dataset size reduced to 13119 genes. Missing values were imputed using the 'impute.knn' function in the R-package "impute". To match with the size of the Discovery set, we kept only those genes that existed in the Discovery set. The final dataset contains 9786 genes.

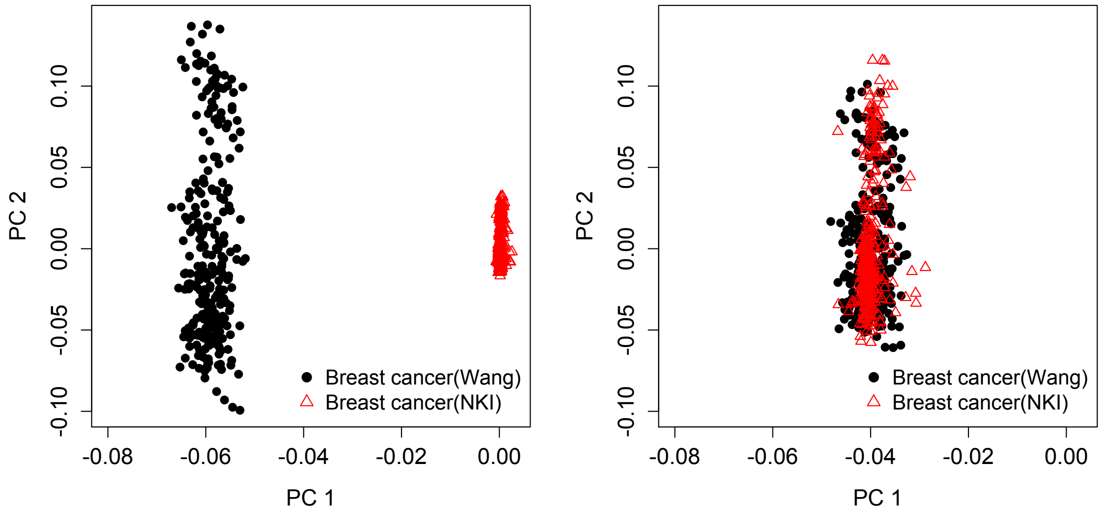

Figure S6: Visualization of the batch effect between the Discovery and Test sets. The combined dataset is projected onto the space defined by the first two principal components before (left) and after (right) undergoing a batch effect correction.

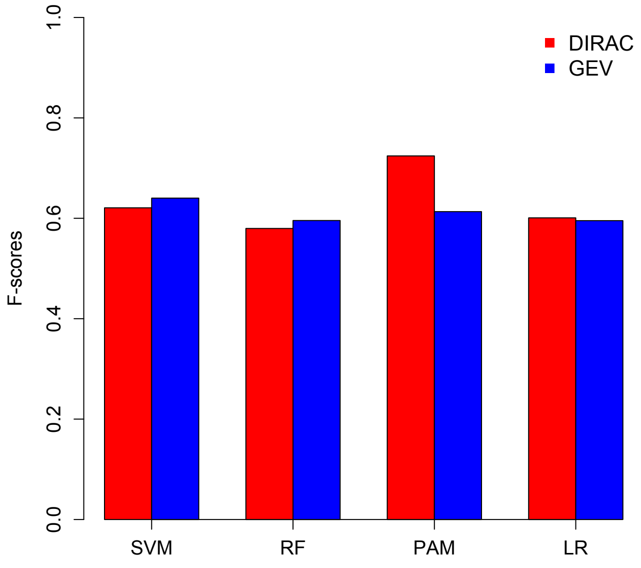

Figure S7: Classification performances of the DIRAC-based and the GEV-based classifiers on the Discovery set. The x-axis shows the classification algorithms used, the y-axis shows the accuracy quantified as F-score.

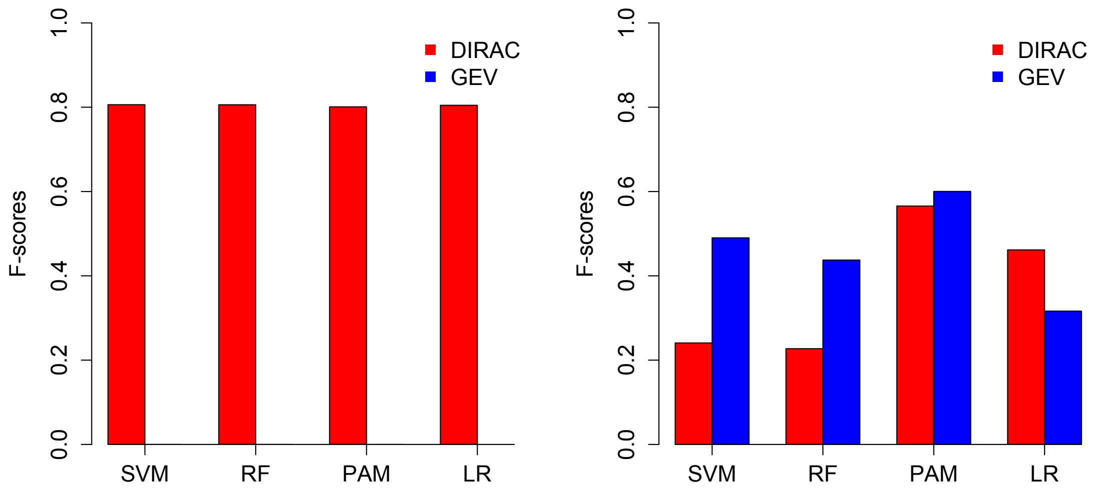

Figure S8: Performances of the gene and pathway signatures generated by the two approaches on the Test set before (left) and after (right) undergoing a batch effect correction. The x-axis shows the classification algorithms used, the y-axis shows the accuracy quantified as F-score.

4.2 The lung cancer dataset

Due to difficulty in finding suitable a dataset that serves as a Test set, here, we only reported results on the discovery set. In this dataset, a binary classifier is trained to discriminate the tumor samples from the normal controls. Since no Test set is present, the results shown here only meant for two ends: first, to support our argument that the DIRAC-based classifier is invariant to the type of classification algorithms used. As shown in Figure S9, the DIRAC-based classifier performed equally across diverse classification algorithms in this dataset as well; Second, to show that a different classifier performance evaluation criteria, other than the F-score we used in this study, show similar result. Figure S10 shows performances of the two approaches when AUC (area under the curve) was used. Clearly, a different accuracy criteria would not change the fact that the DIRAC-based classifier outperforms the GEV-based one.

- **Cancer type:** Inflammatory lung disease.
- **Molecular levels:** Gene expression.
- **Reference:** Bhattacharya *et al.*, *Am J Respir Cell Mol Biol*, 2009.
- **Array platform:** Affymetrix U133 Plus 2.0 array.
- **Number of samples:** 33.
- **Groups to classify:** tumor (n = 15) an controls (n = 18).
- **Usage:** Signature discovery (Discovery set).
- **Availability:** Gene Expression Omnibus (GEO), accession number GSE8581.

- **Preprocessing:** We downloaded normalized probe level data with 54675 probes GEO. We collapsed the probes level data into gene level, by taking the mean of multiple probes that interrogated a single gene. By collapsing into the gene level, dataset size reduced to 21050 genes. No missing values were detected.

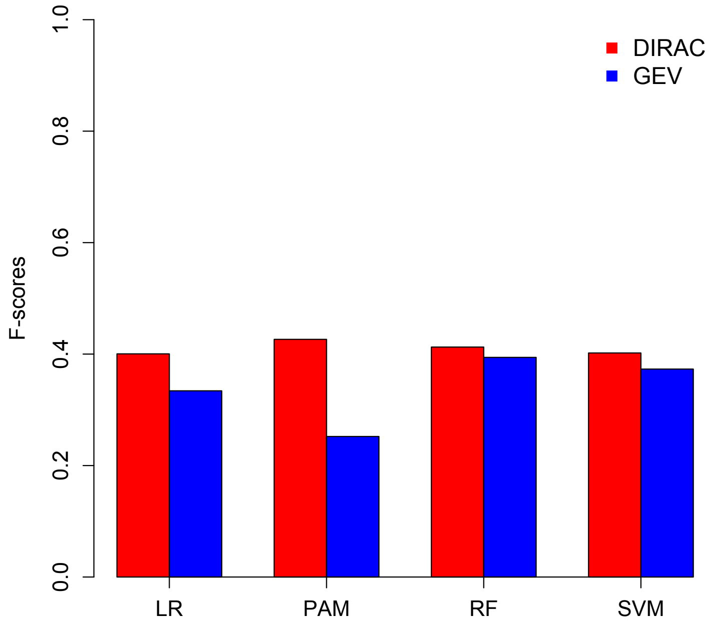

Figure S9: Classification performances of the DIRAC-based and GEV-based classifiers on the lung cancer dataset. The x-axis shows the classification algorithms used, the y-axis shows the accuracy quantified as F-score. The height of each bar present the accuracy obtained on the Discovery set.

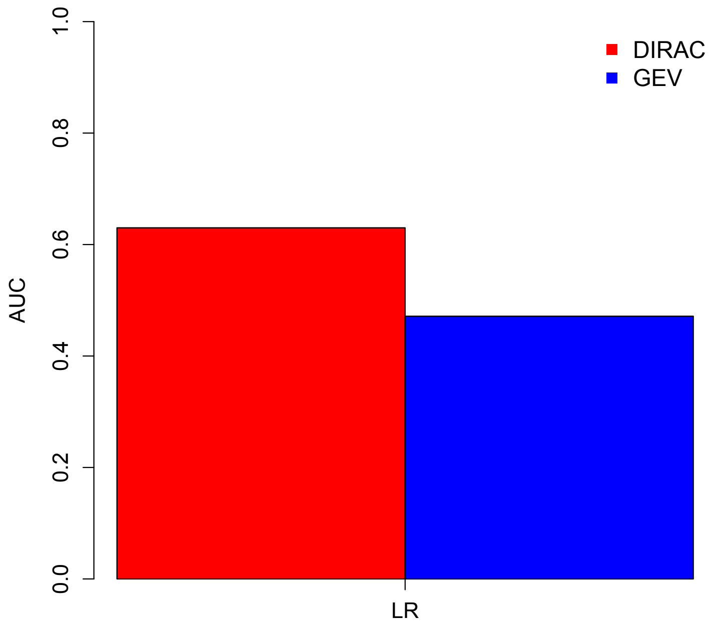

Figure S10: Classification performances of the DIRAC-based and GEV-based classifiers on the lung cancer dataset when LR is used a classifier. The y-axis shows the accuracy quantified as area under the ROC curve (AUC). The height of each bar presents the accuracy obtained on the Discovery set.
